# Broadly Reactive Nanobody Targeting the H3 Hemagglutinin of the Influenza A Virus

**DOI:** 10.32607/actanaturae.27374

**Published:** 2024

**Authors:** D. V. Shcheblyakov, D. V. Voronina, I. A. Favorskaya, I. B. Esmagambetov, I. A. Alekseeva, A. I. Korobkova, E. I. Ryabova, A. A. Derkaev, V. Yu. Kan, A. Sh. Dzharullaeva, A. I. Tukhvatulin, A. S. Bandelyuk, M. M. Shmarov, D. Yu. Logunov, A. L. Gintsburg

**Affiliations:** National Research Center for Epidemiology and Microbiology named after the honorary academician N. F. Gamaleya, Moscow, 123098 Russian Federation; Department of Immunology and Biotechnology, Moscow State Academy of Veterinary Medicine and Biotechnology named after K. I. Skryabin, Moscow, 109472 Russian Federation

**Keywords:** nanobody, single-domain antibody, influenza virus, hemagglutinin, Fc region

## Abstract

Monoclonal antibodies and recombinant antibody fragments are a very promising therapeutic tool to combat infectious diseases. Due to their unique paratope structure, nanobodies (VHHs) hold several advantages over conventional monoclonal antibodies, especially in relation to viral infections. Influenza A viruses (IAVs) remain a major threat to public health. The hemagglutinin (HA) protein is the main protective and immunodominant antigen of IAVs. In this study, three broadly reactive nanobodies (D9.2, E12.2, and D4.2) to H3N2 influenza strains were isolated and Fc-fusion proteins (VHH-Fcs) were obtained and characterized* in vitro*. This modification improved the nanobodies’ binding activity and allowed for their interaction with a wider range of strains. The D9.2-Fc antibody showed a 100% protection rate against mortality *in vivo *in a mouse lethal model. Furthermore, we demonstrated that the observed protection has to do with Fc-FcγR interactions. These results indicate that D9.2-Fc can serve as an effective antiviral agent against the H3N2 influenza infection.

## INTRODUCTION

H3N2 viruses are one of the pathogens responsible for seasonal influenza epidemics; representatives of this influenza A virus (IAV) subtype have been circulating in the human population since 1968 [1]. A seasonal H3N2 infection typically comes with an unprecedented increase in the number of patients with pneumonia that are hospitalized in intensive care units [2] and in individuals with high mortality and complications [3, 4, 5].

Vaccination is one of the most common means used to treat influenza; however, its effectiveness can vary greatly depending on the epidemic season [6, 7]. In addition to the low effectiveness of preventive measures, the activity of modern antiviral drugs has also plummeted due to growing virus resistance [8, 9]. In this context, the development of novel, broadly reactive antiviral drugs and therapeutic monoclonal antibodies (mAb) against influenza becomes crucial. Antigen-binding fragments of *Camelidae *heavy-chain antibodies (nanobodies, VHH) are a promising tool for the early etiotropic therapy of infectious diseases. VHHs present a fully functional domain which binds to an antigen with high affinity and specificity. Nanobodies also demonstrate such outstanding biochemical characteristics as good solubility and thermal/pH stability [10]. Furthermore, VHHs are encoded by a single poly peptide and, thus, can be easily modified: e.g., fused to IgG Fc [11, 12].

The HA glycoprotein is the main immune target. A total of 18 different HA variants are known to date [13, 14]; they form two phylogenetic groups [15]. HA consists of two subunits: HA1 and HA2; these subunits play different roles in the onset of the infectious process. A number of antibodies that specifically interact with H3 HA and the entire phylogenetic group 2 through different mechanisms have been described [16, 17, 18, 19, 20, 1, 22, 23, 24, 25, 26, 27]. The Fc-mediated antibody function is one of the mechanisms involved in combating an influenza infection [28, 29].

## EXPERIMENTAL

**Cell lines**

CHO-S cells were obtained from Thermo Fisher Scientific (USA, cat. No. R80007); MDCK and Caco2 cells were obtained from the Russian collection of vertebrate cell cultures (St. Petersburg, Russia).

**Viruses**

Mouse-adapted IAV A/Aichi/2/68(H3N2) was used.

**Recombinant proteins**

The list of antigens used in the study is presented in *Table 1*.

**Table 1:** The recombinant HA proteins used in in vitro studies

Subtype	Abbreviation	Description	Source	Cat. No.	No. in GenBank/ GISAID databases
H3	H3 HA1 Swiz	HA1 A/Switzerland/9715293/2013 (H3N2)	Sino Biological	40497-V08H1	EPI541659
	H3 HA1	Vic HA1 A/Victoria/210/2009 (H3N2)	Immune Technology	IT-003-00421p	EPI272062
	H3 Swiz	HA0 A/Switzerland/9715293/2013 (H3N2)	Sino Biological	40497-VNAB	EPI541659
	H3 Aichi	HA0 A/Aichi/2/1968 (H3N2)	Sino Biological	11707-V08H	AAA43178.1
	H3 Perth	HA0 A/Perth/16/2009 (H3N2)	Sino Biological	40043-VNAB	ACS71642.1
	H3 Sing	HA0 A/Singapore/INFIMH-16-0019/2016 (H3N2)	Xema	–	EPI1341068
	H3 HK	HA0 A/Hong Kong/45/2019 (H3N2)	–	–	EPI1691930
H4	H4	HA0 A/mallard/Ohio/657/2002 (H4N6)	Sino Biological	11714-V08H1	ABI47995.1
H7	H7 Anhui	HA0 A/Anhui/1/2013 (H7N9)	Sino Biological	40103-V08H	EPI439507
H10	H10	HA0 A/Jiangxi-Donghu/346/2013 (H10N8)	Sino Biological	40359-VNAB	EPI497477

^*^Statistically significant.

**Camel immunization, immune library construction, individual clone selection, and VHH expression and purification**

A Bactrian camel was immunized intramuscularly with recombinant H3 HK HA at a dose of 100 μg. Aluminum hydroxide was used as an adjuvant. Blood (50 ml) was collected from the animal to isolate the peripheral lymphocyte fraction 5 days after the final immunization.

Library construction and specific screening of the clones were performed using inactivated A/Aichi/2/68(H3N2) as an antigen according to [30].

Nanobody expression and purification were carried out as previously described [30].

**Production of VHH-Fc constructs, expression and purification of modified VHHs**

Sequences of the D9.2-Fc, E12.2-Fc, and D4.2-Fc genes encoding the corresponding nanobody fused to the hinge region and Fc of human IgG1 (GenBank: JQ666008.1) were obtained by PCR. The resulting genes were cloned into the pCEP4 vector for eukaryotic expression (Thermo Fisher Scientific, USA). A similar protocol was used to obtain the pCEP4-D9.2-mG2a plasmid encoding the D9.2 nanobody fused with the hinge region and Fc of murine IgG2a (GenBank: V00798.1). To create the pCEP4-D9.2-mG2a LALA-PG plasmid construct, point mutations were introduced into the pCEP4-D9.2-mG2a plasmid by site-directed mutagenesis [31]. Antibodies were expressed and purified as described in [32]. Antibody purity was assessed by Laemmli polyacrylamide gel electrophoresis (SDS-PAGE) under reducing and non-reducing conditions.

The control VHH-Fc–SD36-Fc, corresponding to the nanobody (SD36) to the stem domain (HA2 subunit) of H3 HA fused to human IgG1 Fc, was prepared and analyzed in a similar manner. The amino acid sequence of the nanobody was taken from [33].

**Enzyme-linked immunosorbent assay (ELISA)**

ELISA was carried out according to [32]. To detect the antibodies in the serum of camel, anti-Llama IgG conjugated to horseradish peroxidase (HRP) was used (Bethyl, A160-100P). HRP-conjugated secondary anti-c-Myc (ab1326, Abcam), anti-human IgG, and anti-mouse IgG antibodies (A8667 and A9044, MilliporeSigma, USA) were used to detect the antigen- bound VHHs and VHH-Fcs carrying human and murine Fc, respectively. The half-maximal effective concentration (EC_50_) was calculated using the four-parameter logistic regression in GraphPad Prism 7 (GraphPad Software Inc., USA).

For competitive ELISA, VHH was serially diluted in blocking buffer with a starting concentration of 800 nM (~10 μg/ml). An equal volume of competitive VHH-Fc antibodies (5 nM) was added to wells containing VHH. Bound VHH-Fc was detected using antihuman IgG HRP (A8667, MilliporeSigma, USA). The optical density (OD_450_nm) in the wells containing only VHH-Fc was considered a 100% signal. Inhibition was expressed as the percentage decrease in OD_450_nm in the wells containing the VHH/VHH-Fc mixture compared to the VHH-free wells.

**Western blotting**

Proteins were separated using 10% ready-to-use Mini-PROTEAN® gels (Bio-Rad, USA) and transferred onto an Amersham™ Hybond™ P nitrocellulose membrane (Cytiva, USA). After membrane blocking, VHH-Fc was added to a final concentration of 1 μg/ml. Next, anti-human IgG HRP (A8667, MilliporeSigma, USA) was added. Immunological detection was performed using Clarity™ Western ECL (Bio-Rad) as a substrate.

**Hemagglutination inhibition (HI) assay**

HI assay was carried out according to [34].

**Virus neutralization (VN)**

The VN test in the mode of microneutralization was performed in 96-well culture plates as previously described [35]. Non-neutralized viral particles were detected using rabbit polyclonal antibodies to the NP protein and secondary anti-Rabbit IgG HRP antibodies (Cat: 11675-T62 and SSA003, Sino Biological, China).

The ability of antibodies to inhibit the release of viral progeny from the cell and reduce the plaque size was assessed using the techniques described in [16].

**Evaluation of the prophylactic and therapeutic effectiveness of the antibodies *in vivo***

All the animal experiments, carried out in accordance with Directive 2010/63/EU, FELASA recommendations [36], were approved by the ethical committee of the Federal State Budgetary Institution National Research Center of Epidemiology and Microbiology n.a. N.F. Gamaleya (protocol No. 19 of 2022).

SPF BALB/c mice aged 6–8 weeks, obtained from the Nursery of Laboratory Animals of the Institute of Bioorganic Chemistry of the Russian Academy of Sciences, were used in all the experiments. The animals were infected intranasally with 5 LD_50_ of the mouse-adapted virus A/Aichi/2/68 (H3N2). The animals were observed for 14 days after infection and weighed daily before they were euthanized. Mice that lost 25% or more of their body weight were euthanized.

Detailed information on the antibody administration regimens is presented in the Results section.

Survival was analyzed using the Mantel–Cox method in GraphPad Prism 7 (GraphPad Software Inc., USA).

## RESULTS

**Fig. 1:**
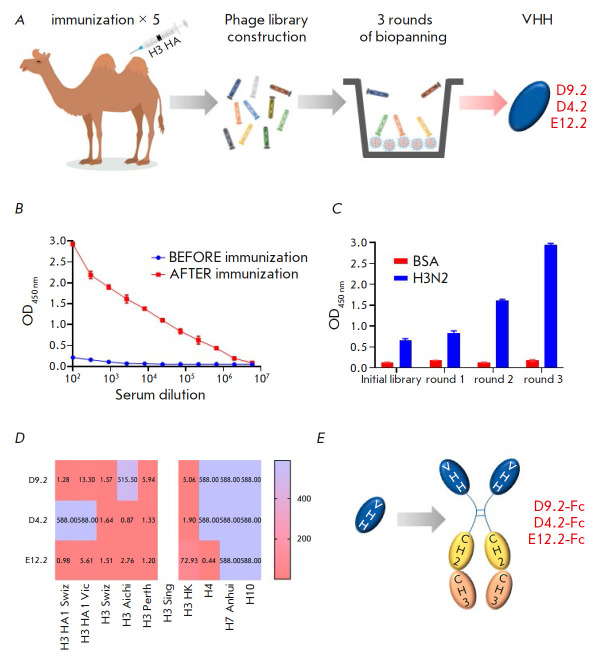
The schematic process of VHH isolation, characterization of the binding activity of the selected VHHs, and their modification: (*A*) – animal immunization and VHH selection; (*B*) – ELISA signals of H3 HA-binding antibodies in the camel serum before and after the fifth immunization; (*C*) – polyclonal phage ELISA: BSA – bovine serum albumin, H3N2 – inactivated A/Aichi/2/1968 IAV; (*D*) – Group 2 HA-binding activity of the selected VHHs detected by ELISA and expressed as EC_50_ values (nM); (E) – strategy for increasing the potency of VHH modified with the Fc region

To collect nanobodies binding to the H3 subtype HA, a Bactrian camel (*Camelus Bactrianus*) was immunized with the recombinant full-length H3 HK HA0 protein previously obtained in CHO-S cells (*Fig. 1A*). The level of HA-specific antibodies in camel serum was monitored by ELISA (*Fig. 1B*). Unlike the control serum, the immune serum obtained after the entire immunization cycle demonstrated specific activity against the H3 HK protein with a binding titer exceeding 1 : 1 500 000. A 1.4 × 10^7^ phage library was constructed using cDNA encoding the VHH sequences isolated from B cells. H3 HA-specific VHHs were selected using phage display by performing three rounds of bio-panning against inactivated A/Aichi/2/68 (H3N2) (*Fig. 1A*). After the third round of panning, significant enrichment of H3N2-specific VHHs was observed (*Fig. 1C*). Of the resulting panel of antibodies, three VHHs (D4.2, D9.2, and E12.2) binding to H3 HK were selected for further studies (*Fig. 1A*).

VHH immunoreactivity was analyzed by ELISA using recombinant HA of the subtypes H3, H4, H7, and H10 (*Fig. 1D*). All VHHs bound to immobilized HA of different H3N2 strains with high affinity, including isolates obtained in 2009, 2013, and 2019. In addition, E12.2 and D4.2 demonstrated affinity for A/Aichi/2/1968 HA. E12.2 also interacted with H4 HA. Both D9.2 and E12.2 recognized the HA1 subunit of HA. Meanwhile, D4.2 did not bind to HA1 but interacted with full-length HA0.

**Fig. 2:**
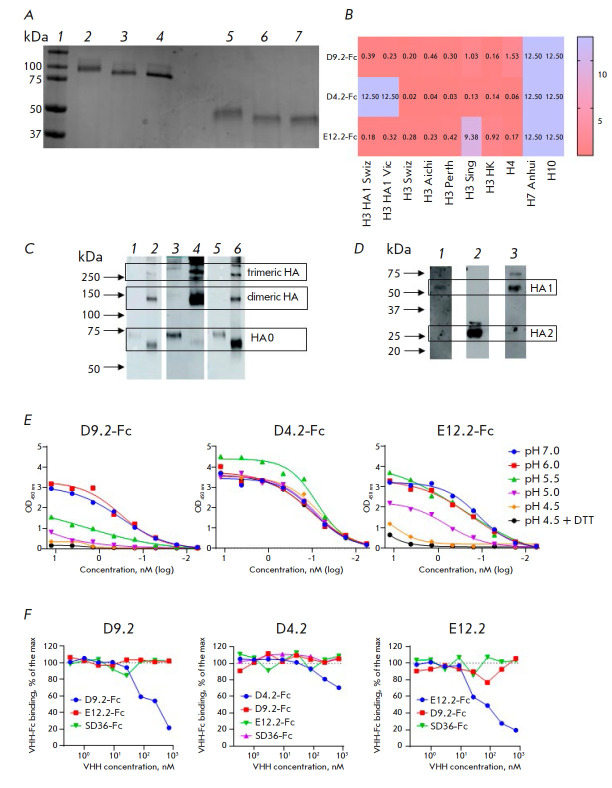
Production of VHH-Fc fusion proteins and their characterization* in vitro*: (*A*) – SDSPAGE of purified VHH-Fc under non-reducing (*2*–*4*) and under reducing (*5*–*7*) conditions: molecular weight ladder (*1*), D9.2-Fc (*2, 5*), E12.2-Fc (*3, 6*), and D4.2-Fc (*4, 7*); (*B*) – binding characteristics of VHH-Fc in relation to different Group 2 HA proteins, expressed as EC_50_ (nM) based on the ELISA assay results; (*C*) – Western blot analysis of the antibody specificity of D9.2-Fc (*1*, *2*), D4.2-Fc (*3, 4*), and E12.2-Fc (*5, 6*) to H3 Swiz HA0 under reducing (*1, 3, 5*) and non-reducing (*2, 4, 6*) conditions; (*D*) – Western blot analysis of VHH-Fc specificity to HA1 and the HS2 subunit of the HA protein: inactivated A/Aichi/2/1968 IAV under reducing conditions, detected using D9.2-Fc (*1*), D4.2-Fc (*2*), and E12.2-Fc (*3*); (*E*) – ELISA demonstrating binding of VHH-Fc to H3 Aichi HA0 cleaved by trypsin-TPCK and incubated in buffer solutions with different pH and DTT; (*F*) – competitive ELISA for identification of VHH-Fc epitopes

In order to increase the activity of the selected nanobodies by natural dimerization, extend the serum half-life, and confer Fc-mediated effector functions, we modified VHH with the Fc region (*Fig. 1E*). The selected VHH sequences were fused into the hinge region and the Fc domain of human IgG1. As a result, the following VHH-Fc constructs were obtained: D9.2-Fc, D4.2-Fc, and E12.2-Fc. Dimerization of VHH-Fc was confirmed by electrophoresis (*Fig. 2A*). The band with a molecular mass of approximately 80–90 kDa under non-reducing conditions corresponds to dimeric VHH-Fc.

The scope of the VHH-Fc binding ability was studied by indirect ELISA using recombinant HA0 and HA1 proteins of different IAV strains (*Fig. 2B*). The introduction of the Fc region in the VHH molecule appeared to enhance the binding effectiveness of each of the VHH-Fc selected, although to a different extent. The most pronounced increase in the affinity was demonstrated for D4.2-Fc: its EC_50_ for H3 Swiz was 22 pM, while the EC_50_ of the monomeric variant was 1,642 pM. Monomeric D9.2 could barely bind to H3 Aichi, while the EC_50_ of the Fc-fusion form for this strain was 0.46 nM. Both D9.2-Fc and D4.2-Fc gained the ability to bind to H4 HA. The least pronounced effect of the Fc modification was shown for E12.2.

Assessment of VHH-Fc specificity by western blotting showed that the selected antibodies recognize mono-, di-, and trimeric HA forms (*Fig. 2C*). Immunoblotting also showed that the antibodies D9.2-Fc and E12.2-Fc specifically bind to the HA1 subunit, while D4.2-Fc has specificity to HA2 (*Fig. 2D*). Next, we analyzed whether the epitopes recognized by the antibodies degrade in decreased pH conditions (*Fig. 2E*). During membrane fusion, HA is known to undergo significant conformational changes due to a decrease in pH in host cell endosomes. Despite the fact that, unlike HA2, the HA1 subunit does not undergo such major rearrangements [37, 38], the activity of HA1-binding antibodies (D9.2-Fc and E12.2-Fc) was reduced with a decrease in pH and completely lost upon DTT addition. This is because the latter eliminates HA1 from HA. However, D4.2-Fc proved to bind equally to HA at different pH values, as well as DTT-treated HA, which confirms that the epitope is located in the HA2 subunit.

Competitive ELISA showed that the three VHH-Fc clones recognize different non-overlapping epitopes on the HA surface (*Fig. 2F*). The HA2-binding antibody D4.2-Fc did not compete with the control VHH-Fc for binding with HA2 (SD36-Fc).

**Fig. 3:**
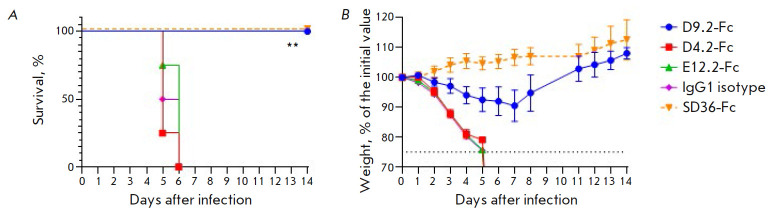
The preventive effectiveness of VHH-Fc *in vivo*: (*A*) – survival curves, only the differences between the control and D9.2-Fc groups are shown (**p = 0.002); (*B*) – body weight curves for surviving mice, data are presented as mean values ± SEM

The protective activity of VHH-Fc *in vivo *was studied using a lethal mouse model (*Fig. 3*). BALB/c mice were administered with 1 mg/kg of VHH-Fc intranasally 1 h before infection. The animals in the control group received the IgG1 isotype: an irrelevant VHH-Fc to the SARS-CoV-2 S protein. The SD36-Fc antibody served as a positive control.

The D9.2-Fc antibody protected 100% of the animals from death. Weight loss in this group did not exceed 10% on average. By the end of the experiment, mouse weight exceeded the original weight. Neither E12.2-Fc nor D4.2-Fc demonstrated protective activity. Therefore, D9.2-Fc was selected for further studies* in vivo*.

**Fig. 4:**
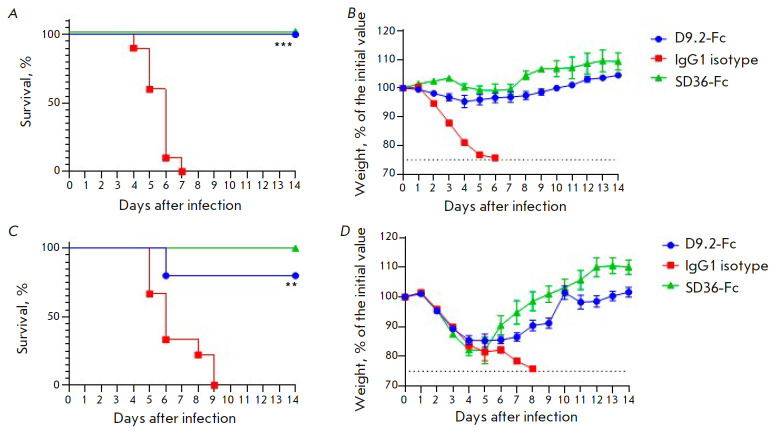
The effectiveness of D9.2-Fc in preventive (*A*) and (*B*) and therapeutic (*C*) and (*D*) regimens *in vivo*: (*A*) and (*C*) – survival curves (***p = 0.0002, **p = 0.0021); (*B*) and (*D*) – body weight curves for surviving mice, data are presented as mean values ± SEM

We further assessed the prophylactic effectiveness of systemic D9.2-Fc administration against a lethal H3N2 infection (*Fig. 4 A,B*). Mice were injected with antibodies at a dose of 10 mg/kg intraperitoneally 24 h before IAV infection. The animals treated with D9.2-Fc showed no disease signs; weight loss was either absent or insignificant. The control mice died after 7 days.

In order to estimate the therapeutic effectiveness of D9.2-Fc, mice were intraperitoneally injected with 40 mg/kg of D9.2-Fc 24 h post-infection (*Fig. 4 C,D*). Mice from the control group died by day 9 after infection. A total of 80% of the animals receiving D9.2-Fc survived; the change in the body weight did not exceed 15%; the weight of all the mice returned to its initial values by the end of the observation period.

To study the mechanism of D9.2-Fc antiviral action, we assessed the activity of VHH-Fc by HI assay and by different variations of VN. The antibody did not inhibit any hemagglutination activity of IAV in the microneutralization assay and did not exhibit virusneutralizing activity in the plaque neutralization test. A study of the ability of VHH-Fc to inhibit virus release from the cell also showed no neutralizing properties by D9.2-Fc.

**Fig. 5:**
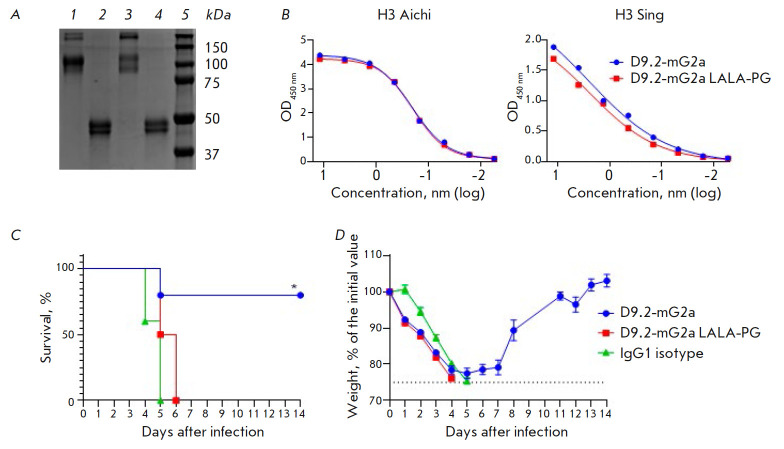
*In vivo *protection by D9.2 is dependent on Fc-FcγR interactions: (*A*) – SDS-PAGE of the resulting antibody constructs under non-reducing (*1, 3*) and reducing (*2, 4*) conditions: D9.2-mG2a (*1, 2*) and D9.2-mG2a LALA-PG (*3, 4*), molecular weight ladder (*5*); (*B*) – ELISA demonstrating binding of the above-mentioned antibodies with H3 Aichi and H3 Sing HAs; (*C*) – survival curves (differences between D9.2-mG2a and IgG1 groups: *p = 0.0361; differences between D9.2-mG2a and LALA-PG groups – *p = 0.0116); (*D*) – body weight curves for surviving mice, data are presented as mean values ± SEM

Since D9.2-Fc did not show any ability to neutralize IAV, we assumed that its effectiveness *in vivo *is due to Fc-dependent effector functions. For this reason, we obtained two additional D9.2 variants: VHH fused to murine IgG2a Fc (D9.2-mG2a) and D9.2-mG2a LALA-PG carrying Fc with the mutations L234A, L235A, and P329G (*Fig. 5*). The LALA-PG mutation complex inhibits binding to FcγR and C1q, while interaction with FcRn and Fc stability remained unaffected [39]. ELISA demonstrated that these mutations do not affect D9.2 binding to HA (*Fig. 5B*). To assess and compare the protective properties of the resulting constructs, we injected mice intraperitoneally with antibodies at a dose of 5 mg/kg 24 h prior to infection (*Fig. 5 C,D*). Mice (four out of five) receiving D9.2-mG2a were protected from death, while all mice treated with LALA-PG, as well as the control mice, died by day 6. Therefore, the Fc-FcγR interaction is required in order to protect the animals in the presence of non-neutralizing D9.2 antibody *in vivo*.

## DISCUSSION

To date, the use of mAbs for infection prevention and treatment has been one of the promising areas of medicine. Nanobodies (VHHs) are considered a reasonable and effective alternative to conventional IgG. The possibility of using VHHs as antibacterial [40, 41] and antiviral antibodies [32, 42, 43] has recently been under active consideration. VHHs consisting of a single polypeptide can be successfully used as a part of adenoviral vectors [44, 45], adeno-associated viral vectors [46, 47], and mRNA [48] for passive immunization. In this work, we identified three VHHs: D9.2, D4.2, and E12.2; these nanobodies are specific to different HA epitopes in H3N2. D9.2 and E12.2 bind to the HA1 subunit, whereas D4.2 interacts with HA2. These VHHs recognize the HA of different H3N2 strains. In addition, monomeric E12.2 can bind to H4 HA.

Enhancement of the VHH antiviral effect by multimerization has been previously reported. VHH P2C5 dimerization has resulted in a 200-fold increase in neutralizing activity against SARS-CoV-2 [49], while the dimer of another anti-S VHH Fu2* A* Survival, % Survival, % Days after infection Days after infection Days after infection Days after infection 100 50 0 100 50 0 0 1 2 3 4 5 6 7 8 9 10 11 12 13 14 0 1 2 3 4 5 6 7 8 9 10 11 12 13 14 0 1 2 3 4 5 6 7 8 9 10 11 12 13 14 0 1 2 3 4 5 6 7 8 9 10 11 12 13 14 *** ** 120 110 100 90 80 70 120 110 100 90 80 70 Weight, % of the initial value Weight, % of the initial value* B* D9.2-Fc IgG1 isotype SD36-Fc D9.2-Fc IgG1 isotype SD36-Fc* C D* Fig. 4. The effectiveness of D9.2-Fc in preventive (*A*) and (*B*) and therapeutic (*C*) and (*D*) regimens *in vivo*: (*A*) and (*C*) – survival curves (***p = 0.0002, **p = 0.0021); (*B*) and (*D*) – body weight curves for surviving mice, data are presented as mean values ± SEM was shown to be 10 times more effective in neutralizing the virus compared to its monomeric form [50]. According to Hultberg A. et al., a 4,000-fold increase in VHH activity can be achieved; this was demonstrated for bivalent VHH, which neutralizes the respiratory syncytial virus [12]. A similar observation was made for Fc-fusion VHH, since the introduction of the Fc region to the molecule results in its natural dimerization [51, 52]. Furthermore, an expansion of the binding spectrum of some VHHs due to multimerization was shown. Bivalent anti-influenza VHH R1a-B6 acquired the ability to neutralize H2N2 viruses [53], while Fc-fusion G2.3 neutralized H5N2 and H9N2 [32]. The Fc-fusion VHH active against SARS-CoV-1 demonstrated cross-reactivity with SARS-CoV-2 [54]. In addition, the Fc modification allows for the recruitment of effector functions, including complement activation and/or antibody-dependent cellular cytotoxicity and phagocytosis, which play a crucial role in combating an influenza infection [29]. Therefore, we fused the obtained VHHs to human IgG1 Fc, and Fc-mediated dimerization resulted in increased binding activity and the ability to interact with H4 HA (for VHH D9.2 and D4.2). However, modification of E12.2 resulted in minimal (compared to other VHHs) increase in binding capacity, suggesting that the potential for enhancing the antibody binding efficiency and spectrum through multimerization depends on the epitope.

We analyzed the effectiveness of the selected antibodies* in vivo *and found that intranasal administration of D9.2-Fc one hour prior to infection fully protects animals from death, while D4.2-Fc and E12.2-Fc do not. Considering these results, D9.2-Fc was selected for further analysis of its prophylactic and therapeutic properties *in vivo*. Systemic administration of D9.2-Fc 24 h prior to infection yielded 100% antibody protection, while antibody injection 24 h after infection resulted in the survival of 80% of the animals.

We also assessed the virus neutralizing activity of D9.2-Fc *in vitro*. However, D9.2-Fc does not have the ability to neutralize H3N2. Thus, we hypothesized that its protective properties *in vivo *depend on the Fc-mediated effector functions of the antibody. The Fc region of human IgG1 is known to be able to bind to murine FcγR [55]. Nevertheless, in certain cases, the IgG subtype plays a crucial role in mAb protection in a lethal mouse model. MAbs with the constant region of the mouse IgG2a heavy chain binding to HA2 and targeting the HA interface were shown to improve protection *in vivo *compared to the original IgG subtype. The reason for this is the higher affinity of the Fc region of the IgG2a subtype for FcγR, compared to IgG1 [56, 57]. Despite the lack of a consensus in researchers’ views on the extent to which the antiviral effect of HA1-specific mAbs is determined by the Fc-mediated functions *in vivo*, there is data confirming at least a partial dependence of anti-HA1 mAb protection on the Fc-FcγR interaction [19, 25, 26, 58]. We compared the protective properties of D9.2 fused to murine IgG2a Fc (D9.2-mG2a) and D9.2 carrying LALA-PG mutations (D9.2-mG2a LALA-PG) *in vivo* and discovered that D9.2-mG2a ensured the survival of 80% of the animals, while the entire group of mice receiving LALA-PG died. Thus, we have established that D9.2-Fc protects animals through the Fc-FcγR interaction.

## CONCLUSIONS

In this work, we identified three VHH clones that recognize non-overlapping epitopes in the HA structure and exhibit activity against the HA of different H3N2 strains. We expanded the VHH binding spectrum by modifying them with the Fc region. Of the three VHH-Fcs selected, only D9.2-Fc demonstrated protective activity *in vivo *in a murine model of the influenza infection. Despite the lack of neutralizing activity against H3N2, D9.2-Fc can provide effective protection *in vivo *through the Fc-mediated mechanisms.

## Abbreviations

IAV: influenza A virus
HA: hemagglutinin
VHH: variable domain of heavy-chain antibody of Camelidae family members
ELISA: enzyme-linked immunosorbent assay
EC50: half-maximal effective concentration
LD50: median lethal dose
mAb: monoclonal antibodies
Fc: fragment crystallizable region
SDS-PAGE: sodium dodecyl sulfate-polyacrylamide gel electrophoresis
HRP: horseradish peroxidase
OD450nm: optical density measured at a wavelength of 450 nm
HI: hemagglutination inhibition
NA: neutralization assay
DTT: dithiothreitol
SEM: standard error of the mean
